# The role of climate change education on individual lifetime carbon emissions

**DOI:** 10.1371/journal.pone.0206266

**Published:** 2020-02-04

**Authors:** Eugene C. Cordero, Diana Centeno, Anne Marie Todd

**Affiliations:** 1 Department of Meteorology and Climate Science, San José State University, San José, California, United States of America; 2 Department of Communication Studies, San José State University, San José, California, United States of America; Universite du Quebec a Montreal, CANADA

## Abstract

Strategies to mitigate climate change often center on clean technologies, such as electric vehicles and solar panels, while the mitigation potential of a quality educational experience is rarely discussed. In this paper, we investigate the long-term impact that an intensive one-year university course had on individual carbon emissions by surveying students at least five years after having taken the course. A majority of course graduates reported pro-environmental decisions (i.e., type of car to buy, food choices) that they attributed at least in part to experiences gained in the course. Furthermore, our carbon footprint analysis suggests that for the average course graduate, these decisions reduced their individual carbon emissions by 2.86 tons of CO_2_ per year. Surveys and focus group interviews identify that course graduates have developed a strong personal connection to climate change solutions, and this is realized in their daily behaviors and through their professional careers. The paper discusses in more detail the specific components of the course that are believed to be most impactful, and the uncertainties associated with this type of research design. Our analysis also demonstrates that if similar education programs were applied at scale, the potential reductions in carbon emissions would be of similar magnitude to other large-scale mitigation strategies, such as rooftop solar or electric vehicles.

## 1. Introduction

In 1992, the United Nations Framework Convention on Climate Change (UNFCC) stated, “Education is an essential element for mounting an adequate global response to climate change” [1]. Few would argue against the importance of education in providing an informed response to environmental problems. Solutions to climate change tend to focus on mitigation and adaptation measures, and successful implementation of either strategy requires an informed and educated citizenry. Interest in education and climate change has increased in recent years [2] in part due to leadership efforts from organizations like the United Nations Education, Scientific, and Cultural Organization (UNESCO) that continue to advocate for educational efforts to respond to climate change [3]. Yet despite the notion of education’s importance in responding to climate change, education is rarely mentioned in discussions of today’s major climate solution strategies. One reason that education programs may not feature prominently in discussions about climate change mitigation is that few studies verify the effective reductions in carbon emissions resulting from education programs. Although several studies have linked environmental education and environmental quality (e.g., Education and water quality [4]; Education and air quality [5]; and Education and energy reduction [6]), the environmental education literature is relatively sparse [7]. And while the potential to reduce carbon emissions through behavior programs is clear (e.g., [8]), connections to education over time have not been as well established [9]. This is in contrast to technologies such as renewable energy generation and the electrification of automobiles that can demonstrate reductions in carbon emissions using more easily accessible data. Should education be shown to be an effective tool to reducing emissions via changes in attitudes and behavior, it would seem likely that funding and interest in such methods would become more widespread and well supported.

Education has been found to be one method for promoting behavior change, but only under certain circumstances (e.g., [10]; [11]). The environmental education literature offers insights into the connections between education and behavior change, and it also provides guidance on how to encourage pro-environmental behavior [12]; [13]; [14]; [15]. The notion that knowledge leads to awareness and then to action has been countered with studies that document that knowledge and skills are not enough to change behavior (e.g., [16]). The literature suggests that more personal factors such as a deep connection to nature, personal relevance to the issue and personal agency towards action are important elements that contribute to successful behavior change programs (e.g., [10]; [17]; [18]; [19]). Even among successful programs, the question of how long the intended behavior is sustained can vary depending on the type of intervention, with longer and more sustained engagements tending to have more long-lasting impacts [20]. This previous research informs educational research programs towards designs that not only focus on information but also promote the personal qualities that can support sustained action.

A growing base of literature is developing around climate change education as national standards move towards inclusion of this subject in the core curriculum [21], and educators negotiate the teaching of this sometimes ‘controversial’ subject (e.g., [22]; [23]; [24]). While there are similarities to the teaching of other environmental topics, climate change includes some unique education challenges that make teaching this topic especially difficult [25]; [26]; [27]. The science is highly complex and spans various areas in the natural and physical sciences, and yet the implications of our changing climate and the role of human activities make this scientific topic both a social and a political issue. Despite the goals of environmental education organizations like the UNESCO, relatively few climate change education programs remain that have successfully demonstrated the type of behavior change needed to effectively respond to climate change [23]; [28]; [29]. Further, even among existing climate change education resources offered in textbooks and through government programs, it appears there are opportunities to promote more effective emission-reduction strategies [30].

The purpose of this paper is to evaluate the impact of an intensive university climate change course on individual long-term carbon emissions. The design of the course is described including the background research framework that was employed to help students develop a deep connection with climate change and climate solutions. Five years of graduates from the course were surveyed at least five years after they took the course. The results of both survey data and focus group interviews provide an indication of the long-term impact of the course, and they contribute to our understanding of the potential role that education can play in long-term behaviors and attitudes. We then quantify the reductions in annual carbon emissions resulting from graduates’ pro-environmental behavior, and we compare the reductions achieved through this education program with other climate change mitigation measures. Additional discussion is provided about the educational approach and the factors we felt were critical to the success of the education program.

## 2. Methods

The San Jose State University IRB committee has approved this human subject research (F15035) and all participants have provided written consent.

### 2.1. University course and students

In fall 2007, a new course was offered at San José State University (SJSU) that satisfied all three subject areas of the upper division general education (GE) requirements, plus the campus upper division writing requirement. The course, COMM/ENVS/GEOL/HUM/METR 168 & 168W: Global Climate Change I & II (hereafter referred to as COMM 168), is taught over an academic year, with six credit hours in the fall semester, and three credit hours in the following spring semester. The course is team taught by three faculty members from different departments with expertise in the core themes of climate science, climate mitigation and environmental communication. Although different professors taught the course during the five-year study period, the syllabus was consistent through the five years. During this same five-year period, student enrollment came from a broad distribution of the campus colleges, as shown in Table 1. The course uses a number of design approaches to impact students in ways that maximize effects on students’ personal and professional lives, and this is described in more detail in section 3, Course Design. COMM 168 has been taught every year since 2007 and continues to be a well-enrolled class at SJSU.

**Table 1:** The distribution of colleges from the reported major for each of the participants.

College	% of students
College of Social Sciences	40%
College of Humanities and the Arts	22%
College of Business	19%
College of Applied Sciences and Arts	10%
College of Science	7%

### 2.2. Survey and focus groups

An 18-item survey instrument (provided in the S1 Text) was developed to study participants’ beliefs about climate change and whether their own personal actions to mitigate climate change could be associated with taking the COMM 168 course. The survey was broadly based on questions about climate change drawn from [31] and [32], and included questions that used a five-element Likert scale (strongly agree, agree, don’t know, disagree, or strongly disagree), multiple choice, and free response. A draft survey was trialed at SJSU by other educators and was revised based on their feedback. Of the more than 500 students who took the course, 104 students from the five different course iterations between 2007 and 2012 completed the survey. We emphasize that the survey was given to students at least five years after they completed the course, and no surveys were given before participants took the course. The categories of questions focused on participants’ a) attitudes and beliefs about global warming and whether they perceive it to affect them personally, and b) whether any of the participants’ current pro-environmental behaviors can be attributed to taking the COMM 168 course. The survey data was collected using an online platform where participant email was used to ensure only one response was collected per participant. Once the data was collected, spreadsheet statistical techniques, including pivot tables, were used to analyze participant data based on responses to different items.

After evaluating the survey responses and noting themes in the utility of the course and personal climate change mitigation strategies, we followed up with focus group interviews to gain more in-depth understanding of the enduring influence of the course on students’ personal and professional lives. Including a qualitative approach, such as focus group interviews, can complement the survey analysis and ultimately enhance the quality of the resulting analysis [33].

Focus group participants were randomly selected from the 100+ survey respondents. We conducted two focus groups with a total of five participants in a classroom at San José State University. Participants were asked a series of open-ended questions about the course and its impact on their current lives. Once the focus group interviews were completed, focus group transcripts were analyzed according to thematic analysis. The goal of a thematic analysis was to identify patterns in the data to bring clarity to the research questions. First, we interpreted patterns in the focus group responses by identifying themes in the transcripts that were common across the interviewees in different focus groups. Then select quotes and phrases were chosen to illustrate the identified themes. These quotes and phrases were woven into a narrative to describe the focus group responses in a coherent way. This exploratory approach to thematic analysis enabled us to present a rich description of student experiences in the course and perceptions of climate change issues. Copies of the survey, focus group scripts, and focus group protocols are provided in S1 and S3 Texts.

### 2.3. Estimating carbon emission reductions from the survey responses

Once responses to the survey questions were obtained, the potential carbon reductions from the decisions made by participants were estimated. Details of the procedure used are provided in S2 Text, but we briefly describe the method here. We use the CoolClimate Calculator [34] an online household carbon footprint calculator that has been well documented and verified in a number of studies (e.g., [35]; [36]; [37]; [38]). The carbon footprint calculator is used to estimate how a particular action attributed to taking COMM 168 would impact individual annual carbon emissions. We start by calculating the annual carbon emissions for an average person in California. Then, based on the response to a particular question (e.g., participant attributed their current purchasing of renewable energy from their utility to the COMM 168 course), we use the calculator to determine the reduction in annual carbon emissions due to that particular action (e.g., participant reduced emissions by 1.38 tons/year by purchasing renewable energy from their utility). This procedure is repeated for each of the actions identified in the survey, and thus allows us to estimate how particular actions have changed individual carbon emissions. We acknowledge that although participants attributed particular actions to the COMM 168 course, other experiences either before or after the course may have also contributed towards these pro-environmental attitudes and behaviors. Our notion is that this intensive one-year class on climate change played a key or leading role in the development of these attitude and behaviors.

## 3. Course design

The COMM 168 course was designed to promote lasting responsible environmental behavior through an educational model broadly based on the environmental education research of [17]. In this research, Hungerford and Volk identified three predictor variables or factors that contribute to pro-environmental behavior. The first factor is labeled as an entry-level variable and describes the importance of an empathetic perspective towards nature and the environment. The second factor is labeled as the ownership variables and describes the importance of both in-depth knowledge about the issue and a personal connection to the issue. The third factor is the empowerment variable, and this describes the understanding and skills around solutions to the issue, together with a sense of personal agency. As described in various later studies (e.g., [10]; [39]; [40], these three factors are important components to successful behavior change educational programs.

**Fig 1:**
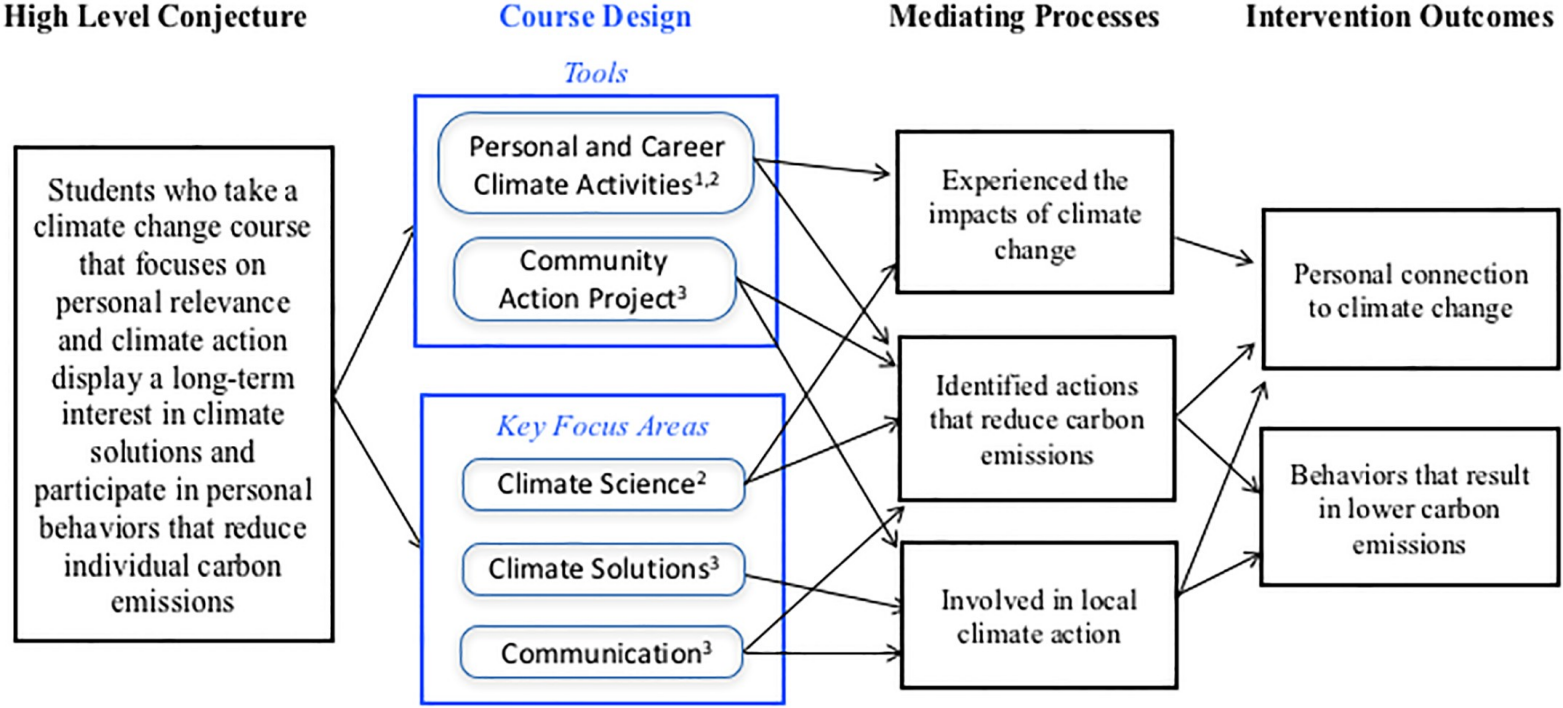
Conjecture map illustrating the connection between the course design elements, the mediating processes (e.g., observable interactions) and the intervention outcomes. In the course design column, the item superscripts indicate an alignment with predictor variables (i.e., 1—entry level; 2—ownership; 3—empowerment).

To illustrate the theoretical connection between the design elements of the course and the expected outcomes, we use a conjecture map in Fig 1 [41] to illustrate what we believe are the most salient connections between the primary conjecture, key elements of the intervention design, the measurable mediating processes and the intervention outcomes. This framework outlines the intermediate processes that support learning, and offers opportunities to measure the effectiveness of these mediating processes towards the intervention outcomes.

The course design includes two primary tools that aim to provide students with the key learning experiences that will lead to the intended outcomes. The first tool is a series of activities where students explore connections between their personal and professional lives and climate change. The second tool is the community action project, where student teams design and implement plans to reduce carbon emissions in a community of their choice. Each of these tools, together with other learning experiences in the class, are structured around the three key focus areas of climate science, climate solutions and communication. Examples of the primary tools followed by the mediating processes are provided below.

The COMM 168 course used a series of activities to help students develop a stronger connection to climate change and to leverage the predictor factors that have been found to promote behavior change. We provide three examples of learning activities that leveraged each of these predictor factors. In one activity focused on careers, students write a paper, supported by research, about the importance of climate change in their specific discipline. The audience of the paper are peers in their field, and students identify at least three reasons why climate change would be important in their discipline. This career activity is most closely aligned with the ownership variable. In another activity focused on individual action, students use an online calculator to compute their own carbon footprint based on their lifestyle, and then they develop a plan for how to reduce their carbon footprint by 10%. Students then implement their carbon reduction plan for a week and report on their experiences. This activity is most closely aligned with the empowerment variable. In a third activity, students participate in a multi-day United Nations (UN) climate negotiation simulation, where students play the role of a delegate representing a specific nation or bloc of nations. This activity provided students with unique perspectives on the impacts of climate change on vulnerable communities, and this activity was most strongly associated with the entry-level variable.

The other primary tool used in the course design is the community action project (CAP), a year-long culminating experience that threads through the two semesters. In the CAP, student teams build on their course knowledge to develop, design and implement projects that respond to climate change in local communities. During the first semester, student teams are formed and develop proposals for their community action project, while in the second semester, student teams are focused on developing and implementing their projects. Examples of CAPs include developing community gardens in the local neighborhood, presenting climate lessons in schools, and creating campaigns to help individuals and businesses move towards some type of climate action. At the end of the second semester, a panel of external judges comprising local government and industry award prizes to the teams with the most innovative and successful projects. The CAP allows students the opportunities to apply their learning in a way that is meaningful and impactful, and there is strong alignment between CAP projects and the predictor variables described above.

Supporting these two instructional tools are the three key focus areas of climate science, climate solutions and environmental communication. For the focus area of climate science, the instruction provides an understanding of the natural and anthropogenic factors that affect the Earth’s climate. Students study the past climate to understand natural factors, and then they focus on the current climate where human activities are the dominant contributor to contemporary changes. Tools like radiative forcing and climate models are used to help students identify evidence connecting human activities and climate change.

For the focus area of climate solutions, students study how both policy mechanisms and personal actions can help mitigate climate change. Through various case studies, students look at the role that local, state and national policies can have on improving environmental conditions. Related issues such as environmental justice and the slow uptake of climate action in government are also discussed. Other areas of climate change mitigation include studies of personal behavior around subjects like food, transportation and home energy use.

For the focus area of environmental communication, students look at marketing and communication strategies and the ideas around framing for particular audiences. Students study various media campaigns and develop experience creating their own communication tools designed for a particular audience. A component of this also focuses on analyzing the current public discourse around climate change and how various stakeholders play a role in shaping these discussions.

As referenced in the conjecture map of Fig 1, these course design elements support a number of mediating processes that ultimately can lead to actions and behaviors that reduce carbon emissions. Aspects of the mediating processes and intervention outcomes can be measured using various tools. In this study we have used surveys and focus group interviews to explore students’ knowledge and attitudes about climate change at least five years after completing the course.

The design elements of the course were developed to achieve the stated outcome of developing a personal connection to climate change and participating in behaviors that reduce carbon emissions. As is the case in many educational settings, along the way faculty made adjustments to the course and their teaching to help promote student engagement. However, the primary course design tools and key focus areas were constant throughout the five study years. A copy of the original syllabus is provided in S4 Text.

Finally, when developing this course more than 10 years ago, we were focused on creating a contemporary and action-based learning experience. Only later did we realize that this learning environment was creating unique outcomes, worthy of further study. Although it would have been preferable to have also collected data before and during the course experience, the type of longitudinal analyses presented here is rare in environmental education, and our methodology, although subject to some limitations, provides a unique opportunity to investigate the long-term role of education on personal behavior.

## 4. Results

As described in Sections 2.2 and 2.3, we use surveys and focus groups to study the attitudes and behaviors of graduates of COMM 168 after more than five years following the course completion. These results are analyzed in the below sections.

### 4.1. Survey

The first part of the survey focused on participants’ attitudes and beliefs about global warming. A large majority of participants (83%) agreed with the statement, “Most scientists think global warming is happening.”, and most participants (84%) also felt that global warming would affect their lives “a great deal” or “a moderate amount.” This is notable since the general public often discounts the impacts that global warming will have on them personally [42]; [43]. Most participants (84%) also strongly agreed or agreed with the statement, “I have personally experienced the effects of global warming.”, and when asked about how global warming will affect future generations, 91% said “a great deal.” Because these results are quite different from the average U.S. general public (e.g., [44]), this suggests that the course may have had an influence on students’ long-term beliefs about climate change. Even so, we cannot rule out the possibility that a socially-agreeable bias may be present in participant responses, as described further in the Section 7.

The second group of questions asked about personal actions to reduce climate change and whether the COMM 168 course had any effect on those actions. The general areas of climate action included waste reduction, home energy conservation, transportation and food choices. Each question asked participants to reflect on how participation in COMM 168 may have affected their actions today in those areas.

**Fig 2:**
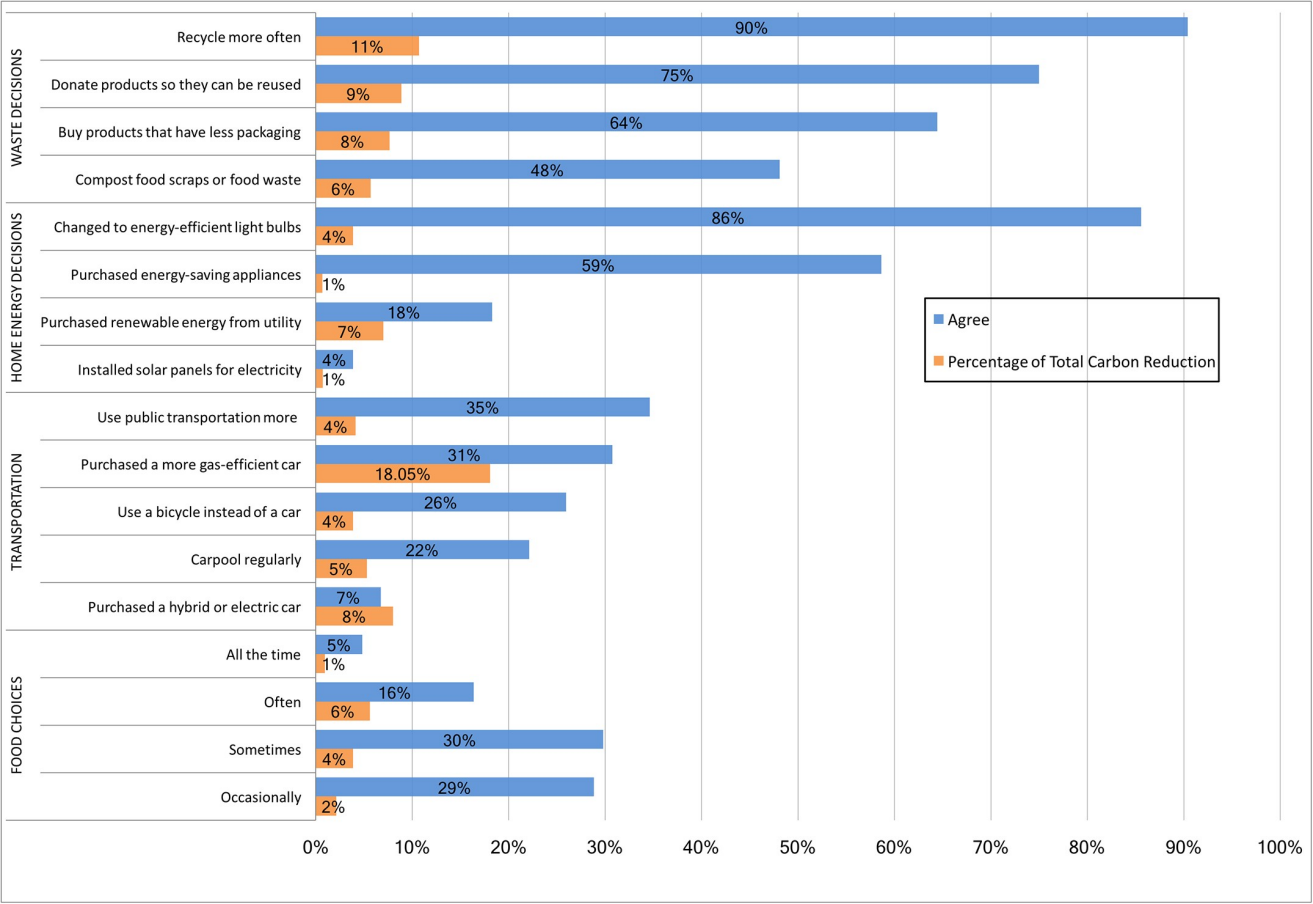
Survey results for questions related to how often participants make choices to reduce carbon emissions as a result of the COMM 168 course. The blue bars represent the percentage of students who agreed with the survey response, while the orange bars represent the impact in carbon emissions in percent relative to the total reductions.

A summary of the results for the different categories is provided in Fig 2. In the waste and home energy conservation categories, a large percentage of participants described engaging in some actions to reduce waste or reduce energy use in their home that they attribute to taking the COMM 168 course. This included recycling more often (95%), changing to more energy efficient light bulbs (86%), giving away or donating products so they can be reused (75%), buying products that have less packaging (64%), and purchasing energy-efficient appliances (59%). Fewer participants reported actions such as composting food scraps (48%), purchasing renewable energy from their utility (18%) and installing solar panels (4%).

In the transportation category, about 25% of participants reported some behavior to reduce emissions that is attributed to the COMM 168 course. This included using public transportation more (35%), using a bicycle for transportation (26%) and carpooling regularly (22%). And in the food choices category, most participants (80%) reported that at least occasionally they made food choices based on reducing carbon emissions.

The survey responses reported here suggest that participant behavior was influenced by the COMM 168 course in ways that continue to impact daily life. The types of actions studied here can be divided into two groups: one-time actions and recurring actions. For example, the purchase of an energy-efficient light bulb or automobile is a one-time action, and these decisions will shape energy use for years into the future. In contrast, recurring actions such as recycling or food choices are made every day, and thus require more consistent engagement or behavior response. In reality, pro-environmental behavior includes both types of actions, and their impacts on carbon emissions can vary depending on the type of action and whether recurring actions become part of an individual’s lifestyle. Given the number of years that elapsed between the course and the survey, the survey provides a glimpse into behaviors that have likely become habitual. In the waste and food categories, some recurring actions were noted by most participants. Although recycling may be viewed as a fairly common action in many Californian communities, food choices and the connection with carbon emissions is not as widely known by the general public (e.g., [30]; [31]). Given that 80% of participants reported some changes to their food choices, it appears that the course did have an impact on decision-making in this category even years after the course.

#### 4.1.1. Estimated carbon emissions

Using the survey responses about the actions that participants took, we estimate the reductions in carbon emissions for all participants using a household carbon footprint calculator. Fig 2 also shows the contribution of each of the survey questions to the total reductions in carbon emissions. While changes to behavior around reducing waste and energy conservation at home were the most common actions taken, the largest reduction in participant-averaged carbon emissions came through transportation decisions. For example, while only 31% of participants reported purchasing a more gas-efficient car, this single action accounted for 18% of all carbon emission reductions observed. In contrast, while over 90% of participants reported that they recycle more often, the combined reduction in carbon emissions only accounted for 11% of the total reductions.

**Fig 3:**
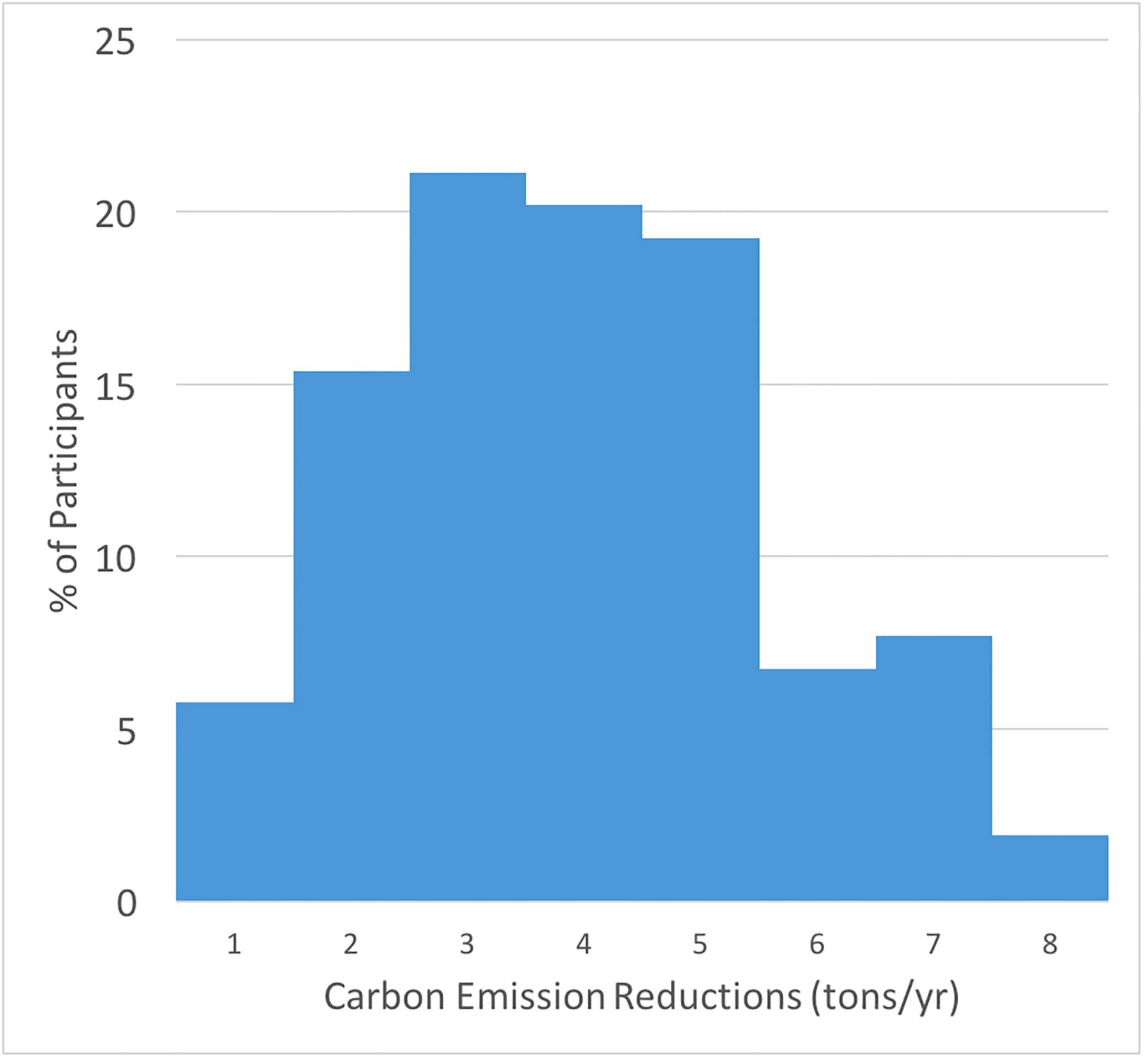
The distribution of carbon emission reductions (tons/year) for participants (n = 104) as a result of the COMM 168 course.

As shown in Fig 3, the average reduction in carbon emissions based on the participant survey responses is 3.54 tons of CO_2_/year, with most participants between 2 and 5 tons of CO_2_/year. About 5% of students reported almost no change (0–1 ton of CO_2_/year), and about 10% reported between 6 and 8 tons of CO_2_/year. Of the four primary categories of carbon emission reductions, changes in transportation were responsible for 40% of the total carbon emission reductions, while waste reduction, food choices and home energy contributed 33%, 13% and 12% respectively of the achieved total carbon emission reductions.

#### 4.1.2. Understanding how personal relevance and carbon emissions are related

**Fig 4:**
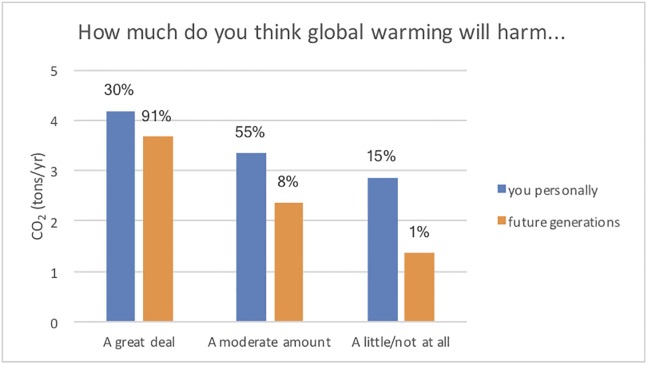
The reductions in carbon emissions (in tons/year) for groups based on their responses to the two questions about how global warming will affect them personally or future generations. The percentage of the total responses for that question is also given above each bar.

Given that one of the goals of the course is to help students develop a personal connection between global warming and their lives, we explore the connections between participant beliefs and total carbon emission reductions through analysis of grouped data. In Fig 4, we show the relationship between individual carbon emission reductions with personal beliefs about how global warming will influence them or future generations. We find that participants who believe that global warming will harm them personally, or will harm future generations, have larger reductions in carbon emissions compared to participants who do not believe there will be a strong impact on them or future generations. Thus, it appears that in most cases, participants were at some level influenced by how they perceived the impact of global warming on their own well-being, or the well-being of future generations, when making personal decisions related to the environment.

**Fig 5:**
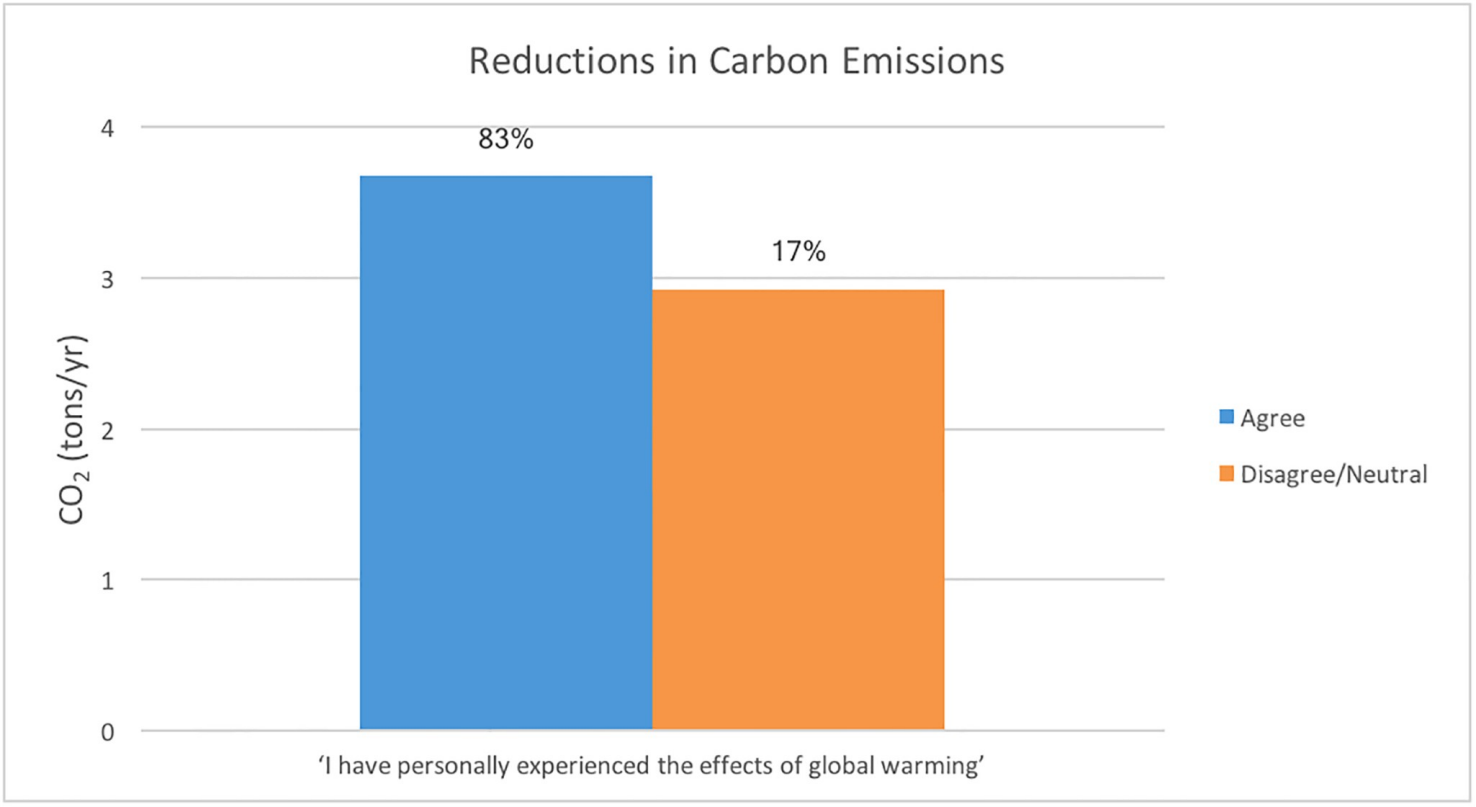
Relationship between statement response and total carbon emission reductions, where agree means strongly agree + agree, while disagree/neutral means strongly disagree + disagree + neutral.

Further, of the participants who agreed (strongly agreed or agreed) to the statement, “I have personally experienced the effects of global warming.” their reductions in carbon emissions were 3.7 tons of CO_2_/year, while for the participants who did not agree with that statement (disagreed, strongly disagreed or neutral), their reductions were only 2.9 tons of CO_2_/year (see Fig 5).

### 4.2. Focus groups

Responses from focus group participants converged around two themes: the importance of daily decisions to mitigate their climate change impact and the importance of engaging their community through climate change communication. Examples of these themes from focus groups responses are provided below, together with relevant connection to the three predictor variables used to inform the course design.

#### 4.2.1. Impact on daily decisions

A hallmark of conversations with graduates from the course was the consideration of climate in daily decisions. Fundamentally, focus group participants recognized the pervasiveness of climate change. As Tara, a focus group participant, noted, “Almost every activity we choose can affect [climate change] in some way, whether we choose to take the bus or drive to work or whether we choose to buy food that’s grown on land that was cleared from rainforests….Since it is in every aspect of our life pretty much, that automatically makes it relevant to all those different aspects.” Other participants agreed and described daily actions that centered on transportation, waste and food choices. Melissa noted, “I think about it all the time…. Definitely how I think about and go about my days, making decisions, even just from using plastic.” And Elaine commented about buying a car after she paid off her student loans “I ended up choosing a Prius C for a lot of reasons. At the time it was pricey, but it just seemed energy efficient. It had what I was looking for while still being helpful for the environment.” These responses exemplify a common theme in the group—the knowledge of climate change gained in the course prompted them to think about the impact of their actions.

The focus group participants noted that they go out of their way to take action because they feel as if they are making a difference. Billy noted, “When everyone does something to mitigate climate change, it will have a huge impact.” Tara concurred, “Almost everything I do can affect the climate somehow. If you start realizing how everything ties together, then pretty much everything you do, every choice you make can affect it in some way.” She continued, “I think every small step does make a difference…. One little step at a time; it all adds up. I’d like to think we’re making a difference. I feel like I am when I contribute a little bit.” Participants suggested that the interdisciplinary focus of the course allowed them to see the connections between their actions and broader climate forcings.

Participant comments demonstrate that environmental actions are not just because of sacrifice but that people feel good about taking action. Lolitta explained, “So when we started the global climate change class, for a week we had to do something eco-friendly. I’m like okay, I’m gonna be a vegan. And I did it totally wrong. I just ate vegetables and fruits all day, and I was starving. But it got me to become vegan, and for a couple years I was. Now I’m a vegetarian, and I’ve stuck to it. I feel good about how I’m living my life, and I’m excited by all the changes that I’m making, and I will continue making these changes because they make me feel great.” Billy noted proudly that he acts because “it’s like a moral obligation.” Ultimately, participants concurred that daily actions matter, and they cited this belief as the reason they continue to take actions. Their comments suggest they are empowered to act because they see themselves as part of the solution.

#### 4.2.2. Community engagement through communication

Overall, focus group participants noted that this course helped them develop experience communicating with other people about their actions and why they are taking them. Participants cited the community action class project as a key element in their understanding of the impact of community engagement. Billy described the lasting impact of “the hands-on approach” of the project: “Those experiences, I think for me, I carry those longer than / more than being in the classroom…. Being with people, doing something that’s going to translate into what I have to do work-wise in the future, [the project was a] translatable experience to the workforce.” Melissa described the lasting impression of the project as a crucial aspect to seeing the impact of action: “It actually bridged the gap between the course and what the community itself is doing.”

Participants noted the impact of the course on life beyond the home. One focus group participant, Elaine, is a manager at Walmart, and she credited the course for her “awareness in an industry with high consumption…. It’s so interesting how much I’ve been able to use just from this course.” She noted her focus as a manager is how to “reduce your inventory, reduce the waste, sell what you need to.” Elaine views Walmart’s waste issues both as a climate issue and a management problem: “I see the huge amounts that they’re throwing away because they’re not managing their business correctly, because they’re not managing their production versus what they need, what they don’t. So that’s one of the things that I work on.” Elaine’s comments exemplify how many graduates of COMM 168 viewed the importance of taking action.

Billy noted the course explained how to make “big issues” like climate change “resonate with your audience… That’s what I do now.” He explains that in his job at the utility company, Pacific Gas and Electric, one of his roles is communicating about energy issues, “That’s my biggest takeaway from this class: messaging. [I now understand] the importance of communicating about climate change in a way where people who don’t have a background in that subject can understand.” Other participants concurred that the course made them experts in climate change, and they now have to think about how to communicate with people who don’t have such extensive knowledge.

Participants also noted the importance of communicating with others about the actions they take. Tara noted: “It doesn’t really help unless you try to bring it out there. If I only ever walk places, no one will ever know unless I try to let them know why I walk places…. If you’re going to make a point by breaking the rules, you first have to know the rules because otherwise it doesn’t mean anything. If I want to rebel by not using a car, I first have to know that everyone thinks using a car is a normal thing to do.” Participants agreed that talking about their own actions helped in discussing climate change issues with others.

The community action project was a key part of the course in giving students experience outside of class in creating change. It also gave them some agency over this issue. Participants described their attempts to make a difference, both in their personal and professional lives. Participants noted the community action project allowed them to see the importance of communication in the design of their projects. The focus group responses suggest that interdisciplinary education including aspects of communication can give students the skills and experience necessary to create change in their own communities.

The outcome of the themes that emerged from the focus groups are broadly aligned with the methodology outlined in the course design (Section 3) and as described in the conjecture map (Fig 1) In particular, students noted a personal connection with climate change (ownership variable), and they demonstrated specific ways either through personal actions or through communication that they could take action (empowerment variable). The entry level variable, which describes a sensitivity or empathy for the environment, was present in some of the focus group remarks, but did not emerge as a central theme.

## 5. Educational approach

We describe a number of key design elements that stood out as critical to the success of the education program we developed and that have sustained student engagement over many years. These include a) connecting climate science to students’ lives, b) providing students with experience creating change in a community of their choice and c) creating a culture devoted to stewardship and action. We found that these elements of the course helped students to connect with the subject in ways that extended into their personal and professional lives, and are broadly aligned with some of the predictor variables that we used to design the course. These elements were not isolated from each other, or from other important elements of the course, including a solid focus on climate science, climate solutions and environmental communication. These elements are in line with the models suggested by other researchers, including personal relevance and empowerment [16]; [23]. We now review each of these elements in more detail to provide insights into how these ideas may be applied to other educational settings.

### 5.1. Connecting science to students’ lives

Various activities in the course were designed to help connect climate change with students’ lives and align with the ownership variable discussed in Fig 1. One project asked students to reflect on how climate change would affect their personal and professional lives. Another project had students track their personal energy use, and then implement a plan to reduce their energy use in their home using data from their home smart meters. These elements appeared to have some lasting impact, as various focus group participants reflected on how the course materials affected their personal and professional lives.

In addition to the actions that were identified in the survey data, open-ended feedback also revealed that the course affected other major decisions, such as where to live and how many children to have. In fact, two of the participants mentioned their decisions to adopt a child or not to have children were influenced by the course. This implies that at least for some of the students, the course content and the implications of climate change affected their personal lives deeply. It appears that some of the high-impact actions identified by [30], such as having fewer children, did resonate with the COMM 168 students.

In a recent study by [23], a systematic review of the climate change education literature identified themes common in successful programs. One of the primary themes identified was a focus on making climate change personally relevant and meaningful for learners. It is noted that this is also a common practice in environmental education and science education, but as we found in our own work here, it can be made especially meaningful given the personal connection that climate change can have to students’ lives.

### 5.2. Creating change in a community of their choice

Another design element of the course was to provide students with real-world experience creating and implementing an action plan to reduce carbon emissions, an activity aligned with the empowerment variable. The Community Action Project (CAP) was the culminating experience where student teams competed to develop the most impactful community-based project. The goal of the CAP was to give students real-world experience developing solutions to climate change. It was our intention that through this experience, students would not only better understand some of the challenges associated with creating change but also gain confidence that change can happen through well-designed efforts. [45] found that using issue investigation and action training was an effective way to promote pro-environmental behavior. And [46] found that students were deeply affected by their service-learning course even years after the experience. The COMM 168 course was focused around the year-long CAP, and feedback from the focus groups shared how impactful the project was for some of the students, as a majority of the focus group participants mentioned the CAP as the most memorable part of the course. Our conclusion that the CAP promoted engagement and student empowerment has also been recognized in various other climate change education programs as a key element in creating effective learning experiences [23].

### 5.3. Creating a culture devoted to stewardship and action

The third aspect of the course that we feel was important to creating impactful and lasting change was the social norms that were established during the year-long course. Although we did not create any design elements at the start of the course to promote this, we do feel this was an important part of the course success. Among the emerging theories of behavior change, community-based social marketing and the strong impact of social norms have been shown to be effective measures for stimulating behavior change [47]; [48]. We also acknowledge that creating a culture for a course is not easy, but there are effective teaching strategies that can help influence class culture, and we outline some of these here.

1. Encouraging group discussions with different students: We did a lot of group work in class, and with 80–120 students per class, we took special efforts to mix students for their group work. This helped students work with new students and be exposed to new ideas. By giving students some challenging subjects to discuss (i.e., how does climate change affect their current or future lives), or challenging situations (i.e., during a UN simulation on climate change where students represented different countries negotiating a climate treaty), we gave students the opportunity to exchange personal ideas about climate. We felt this helped students see multiple views across the class, and if an emerging interest and dedication to climate change arose through the class, it could spread.
2. Faculty committed to climate action: The faculty who taught this course were all deeply committed to climate change solutions, and they were encouraged to share their own personal and professional journeys towards reducing carbon emissions. And because students got to know the faculty fairly well, given the course was taught over an academic year, students had the opportunity to connect with the faculty at a personal level. For example, when faculty reflected on their own personal challenges in reducing emissions associated with driving or eating, students could relate to this. Role models are important in creating social change, and we suggest that having professors committed to environmental solutions was also a factor in creating a social culture for the class that encouraged pro-environmental thinking and behavior. For example, one of the focus group participants mentioned that as a result of the class culture, her ownership of an SUV grew uncomfortable given her shifting connection to the environment. She admitted to deliberately concealing her vehicle type from the faculty, even though the faculty attempted to create a culture of acceptance without judgement. Later after graduating, this participant purchased a hybrid as her next vehicle. This is an example of the social norms that were established in the class that may have extended to students’ lives outside of school and over time.

## 6. Potential role of education on carbon emission reductions

Given the reductions in carbon emissions calculated in Section 4.1.1 (and shown in Fig 3), we now explore the potential role of education as a climate change mitigation strategy. We start by estimating the participant reductions in carbon emissions compared to a control group. The control group is created by using California’s per capita carbon emissions data as estimated by the California Air Resources Board (CARB) [49]. We choose to use California’s per capita carbon emissions for two reasons. First, we do not have a good way to access course participant’s prior behavior retrospectively, and second, we assume that behavior after graduating from the course could change as students become professionals resulting in a potential dramatic lifestyle change. The California per capita carbon emission data show that by 2014, per capita carbon emissions for the average Californian declined by 0.68 tons/year compared to 2009, the midpoint when students had graduated from SJSU. By contrast, the participants in COMM 168 reduced their per capita emissions by 3.54 tons/year. Thus, if we subtract the emission reductions for the average Californian (0.68) from our participants (3.54), we find that the net reduction above the average citizen is 2.86 tons/year (3.54–0.68 = 2.86).

We now use the net reduction in carbon emissions observed for graduates of COMM 168 to compare the potential role of education as a climate change mitigation strategy with other climate change mitigation strategies. For this comparison, we employ the methodology outlined in Project Drawdown, where 80 different technologies or strategies are evaluated based on the potential to cumulatively reduce carbon emissions by 2050 [50].

**Fig 6:**
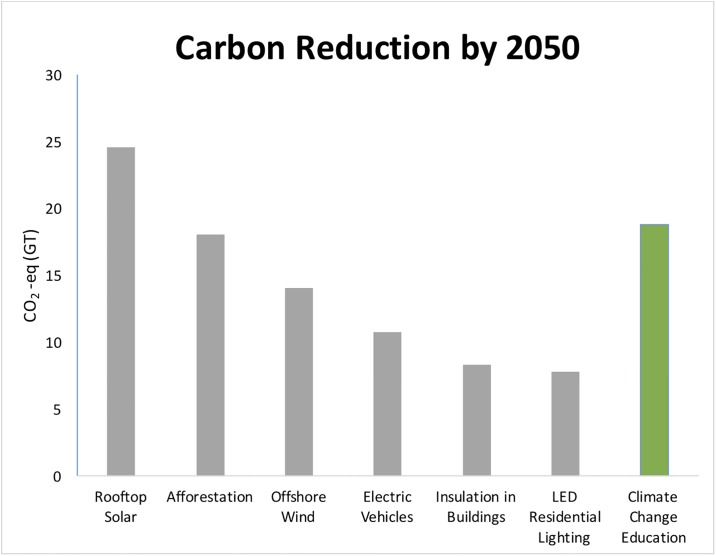
Comparison of various existing technologies that can be applied over a 30-year period (2020–2050) to help reduce global carbon emissions. The potential role of climate change education programs is calculated using the per student carbon reductions estimated from the COMM 168 course.

The following procedure and set of assumptions are used to calculate carbon emission reductions associated with climate change education, as shown in Fig 6. We first assume that a modest investment in climate change education would allow students of secondary school age from middle and high income countries (where their carbon emissions are highest) to receive a specialized climate change education (i.e., using similar educational methodologies as we have described in this paper), and that students who receive this education would each reduce their carbon emissions by 2.86 tons of CO_2_/year (i.e., as in the COMM 168 course), for that year, and for each year following. Further, we assume that such a program would start small at 1 million students and grow by 13% per year until 2050, when the program reaches over 38 million participants. We use 2015 data from the United Nations Educational, Scientific and Cultural Organization (UNESCO) [51] to estimate the number of students of secondary school age from high income and upper middle income as 298 million. This allows us to estimate the percentage of students participating in this specialized climate change education program in 2020 and 2050, assuming the population of secondary students in these countries does not change.

In Fig 6, six of the solutions presented in Project Drawdown are compared with our own estimate for using education as a climate change mitigation strategy. For the solution scenarios developed by Project Drawdown, each of these represents ambitious and yet also technically and economically feasible plans for reducing carbon levels. Technical details and reference literature for all these solutions are presented at www.drawdown.org. As examples, the Rooftop Solar scenario grows the percentage of electricity generated by rooftop solar from 0.4% today to 7% by 2050, while the Electric Vehicles scenario grows the percentage of passenger miles from electric vehicles from less than 1% today to 16% by 2050. For the Climate Change Education scenario we assume that a) each student reduces their carbon emissions by 2.86 tons of CO_2_, similar to the COMM 168 course and b) the adoption of this type of education grows from less than 1% of all secondary students today to 16% of all secondary students by 2050 (note: the number of secondary students is restricted to only high income and upper-middle income countries where residents have higher carbon emissions).

The results of this comparison show that education, if designed appropriately, can potentially be as effective as other established climate change mitigation techniques. Based on the scenario we developed, the implementation of climate change education over a 30-year period (2020–2050) could reduce emissions by 18.8 GT of CO_2_ eq, an amount that would rank in the top quarter (15 out of 80) of the presented solutions in Project Drawdown. Although at scale, the use of education as a climate change mitigation technique is still untested, our analysis suggests that if the educational approach is sound, and if we take the effort to measure the impact of education, we may realize the potential to reduce carbon emissions using education. We also acknowledge that although barriers to developing a successful large-scale climate change education program exist, significant social and political challenges exist with most large-scale solutions to climate change.

## 7. Uncertainties and study limitations

In the following section, we describe a number of uncertainties and study limitations that are important to the interpretation of the results. Although we believe the COMM 168 course provides unique insight into the long-term role that education can have on individual behaviors, especially given the lack of existing studies that look at how education can shape behavior over many years, we also acknowledge the potential limits of such a research design, and thus we are careful here to identify uncertainties and describe limitations in the study. The exposure of such uncertainties and limitations provides the research with a context for interpreting the results and also provides an avenue for researchers to undertake additional studies to investigate the impact that education can have on long-term behavior change.

### 7.1. Uncertainties

The study methodology and analysis include a number of assumptions that contribute to the uncertainties associated with this study, and these are discussed below.

#### Student enrollment

Because the course is titled “Global Climate Change,” students interested in environmental issues may have self-selected into the course. These students may respond more favorably to the course design, and may be more willing to change their behavior in the future given their initial interest in the environment. Although the impact of incentives on bias is not clearly understood [52], the year-long course included a 3-unit incentive where a passing grade in the 9-unit course provided students with an additional 3 units of general education credit. We heard that many students reported that they signed up for the course because of the extra requirements satisfied. Further, we note that when this incentive was removed from the course design in 2014, the initial broad distribution of majors who enrolled in the class declined quite dramatically. In the earlier years with the 3-unit incentive (2007–2013), four colleges (i.e, Social Sciences, Humanities and the Arts, Business, and Health and Human Sciences) each had at least 10% of the registered students. However, once the 3-unit incentive was removed in 2014, only two colleges (i.e., Social Sciences, Humanities and the Arts) had significant enrollment (i.e., more than 10% of students), with the course now having more enrollment from Environmental Studies and Communication Studies. The 3-unit incentive thus appears to have been effective in drawing students from across campus, and this suggests that the course topic was not the only reason students enrolled in the course.

#### Energy calculations

The household carbon footprint calculator was used to estimate how student responses would impact carbon emissions. Although the calculator has been used in a number of studies, various assumptions were made as described in Table 1 of S2 Text. It is clear that some of the carbon reductions attributed to the course experience may have inherent uncertainties. For example, actions such as carpooling regularly, making food choices to reduce emissions and buying energy-star appliances all suggest actions to reduce emissions, and yet the actual reduction amount depends on specifics of the action that are difficult to obtain without a more detailed survey tool. In contrast, the goal of this analysis was to document actions attributable to the course and develop a practical methodology for estimating the carbon reductions using the best tools available.

#### Behavior changes

Another uncertainty that this research only partially uncovered was the motivation for the reported changes. Did participants make lifestyle changes because of environmental concerns or for other reasons, such as financial considerations or ethical concerns? In our focus group, participants reported that pro-environmental outcomes were the primary reason for their choices, but we do not know if this was also the case with all students. Further, without a more detailed survey, it is difficult to understand whether other factors (e.g., social circumstances) also contributed to these changes. This is one reason we chose to use the California per capita emission reduction as a control group, so that pro-environmental trends seen throughout California could be accounted for.

#### Other considerations

We also acknowledge a number of other uncertainties in the design of this study. Participants were surveyed at least five years after taking the course, and we recognize the limits of human memory may skew some of their responses. There may be students who incorrectly remember aspects of the course, and this may have influenced some of our conclusions. This is in part why we chose to do a focus group to more accurately investigate aspects of the course that may have been important.

### 7.2. Study limitations

One limitation in this study is the lack of a control group or a pre-survey. We acknowledge that without such accompanying data, determining the precise relationship between students’ participation in the course and their current attitudes and behaviors is difficult. We did attempt to control for how pro-environmental behaviors in California have become more common over the last decade, but we do not have any data that measured student attitudes or behavior before taking the class. Although further studies should consider the various ways to measure changes in participant attitudes and behaviors, measuring such changes over many years remains a challenge.

Another limitation in the study is the potential for selection bias. Although we attempted to determine whether students self-selected into the course based on their environmental leanings or the 3-unit incentive, we do not have independent data to quantify the role that selection bias had on student enrollment. If students did select this course because of their initial interest in environmental stewardship, this could bias the outcomes of the study.

Another concern is related to biases in participant responses to survey and focus group questions. We acknowledge that a socially-agreeable response bias with regard to behaviors being attributed to the course may exist in the participant responses to surveys and focus group questions. Although we took measures in our survey design and focus group protocol to reduce such biases, it cannot be ruled out that such self-reporting response biases may be present and could influence the reliability of the results.

Finally, we recognize that among the uncertainties identified in section 7.1, none of them have been adequately quantified. Although some of these uncertainties, such as the reliability of the carbon footprint calculator and the related carbon emissions, probably would not influence the primary outcomes of the study, other uncertainties such as initial attitudes of participating students may have a larger influence on the study results.

As we have generally described, establishing linkages between an educational campaign and long-term behavior can be challenging. Other studies that attempt to establish causal links between education and environmental quality also faced similar challenges (e.g., [5]), and yet the insights gained from such work provide a strong motivation for environmental education and this type of research [7]; [53]. Our work is similar. Despite the limitations we have identified, our analysis provides important insights into understanding the role that well-designed climate change education can play on long-term attitudes and behavior.

## 8. Conclusions

The potential role of education on individual carbon emissions was studied using data from students who completed an intensive university course on climate change. Students were surveyed at least five years after having taken the course, and their responses were used to provide both qualitative and quantitative measures of the impact of the course on their attitudes and behavior regarding solutions to climate change. The university course was designed to be impactful, including various elements from the environmental education literature to engage students around personal and social activism. In open-ended feedback and the focus group interviews, students recounted how the course has changed their lives, both personally and professionally. Examples of personal changes included the type of car they drive and the type of food they eat. Examples of professional changes included how they create environmental benefits through their job. The results from the survey data also suggest that the course was impactful, even many years later. Student behavior related to waste decisions, home energy decisions, transportation and food choices all showed significant behavior change that was attributed to the COMM 168 course, and these changes were quantified using a reputable online carbon emissions calculator. The estimated reductions in carbon emissions attributed to the COMM 168 graduates are 3.54 tons/year, compared with the carbon emissions for an average California resident of 25.1 tons/year. It was found that the participants who had personally experienced the effects of global warming, or felt that global warming will harm them personally, had the largest reductions in carbon emissions. Although a number of studies have established links between educational programs and environmental quality, such as water or air quality [7], far fewer studies have established causal links between education and carbon emissions [5].

This study suggests that the design of the COMM 168 course provides elements of the three crucial factors that [17] identify as contributing to pro-environmental behavior: entry-level, ownership, and empowerment variables. Surveys and focus group interviews reveal that graduates of the course feel a lasting personal connection to the issue and have confidence in the success of their actions. This strong sense of personal obligation and the perceived individual agency to address climate change suggest that education that leverages these design elements including community engagement may provide a public benefit. The authors also note that social norms, established through a year-long course and emphasized through various classroom activities, also may have contributed to students’ pro-environmental attitudes and behaviors. However, while previous studies have demonstrated that factors such as having a personal connection (e.g., [23]) and perceived self-efficacy (e.g., [54]) can influence individual behaviors, we acknowledge that other factors are also likely important (e.g., [55]), and understanding how these factors contribute to individual behavior change is complex [39]; [56]. We also acknowledge that there may be cases where structural factors, such as size of home or distance of commute, may obscure the intentions of pro-environmental behavior [57].

The potential to use education as a climate change mitigation measure would be valuable and in line with other mitigation measures if such reductions as achieved in the COMM 168 course could be achieved in other classrooms. We illustrate this through comparisons with other climate change solutions, and show that at scale, climate change education can be as effective in reducing carbon emissions as other solutions such as rooftop solar or electric vehicles. The notion that education is an important part of responding to climate change is not novel (e.g., [29]; [58]), and yet rarely has it been quantified and measured [53]. This paper sheds light on how such measurements could be taken, and it offers a pedagogical insight for how to make education an effective climate change mitigation strategy.

At present, the authors are using similar design approaches to develop a comprehensive science curriculum focused around environmental stewardship and climate action (e.g., [59]; [60]) for middle schools. The Next Generation Science Standards now emphasize applying integrative science fields to solving real-world problems, and this serves as an ideal platform for applying the type of educational platform developed in COMM 168 towards a broader science curriculum for schools. The middle school science curriculum [61] is currently being used in a number of school districts in California, and studies examining changes in student attitudes and behavior will be reported in the future.

## Supporting information

S1 Text Survey instrument used for the graduates of the COMM 168 course. (DOCX)

S2 Text Procedure for estimating reductions in carbon emissions from the survey responses. (DOCX)

S3 Text Focus group protocol. (DOCX)

S4 Text COMM 168 syllabus. (PDF)
