# Supplementary material for: The role of climate change education on individual lifetime carbon emissions
Source: PLoS One. 2020 Feb 4;15(2):e0206266. doi: 10.1371/journal.pone.0206266 (PMC6999882; doi:10.1371/journal.pone.0206266)
Supplement: S1 Text — (DOCX) [file pone.0206266.s001.docx]

S1 Text: Survey instrument used for the graduates of the COMM 168 course.

Intro: Thank you for agreeing to take our survey about the Global Climate Change Course

(COMM/ENVS/GEOL/HUM/METR 168—hereafter COMM 168). This is divided into two

sections: general information and post-course life.

General Questions:

1. What academic year did you enroll in COMM 168?
   1. 2007-2008
   2. 2008-2009
   3. 2009-2010
   4. 2010-2011
   5. 2011-2012
2. What is/was your major?
3. When did you graduate from SJSU?
   1. Spring 2008
   2. Fall 2008
   3. Spring 2009
   4. Fall 2009
   5. Spring 2010
   6. Fall 2010
   7. Spring 2011
   8. Fall 2011
   9. Spring 2012
   10. Currently enrolled
4. How much do you think global warming will harm future generations?
   1. Not at all
   2. Only a little
   3. A moderate amount
   4. A great deal
   5. Don’t know
5. How much do you think global warming will harm you personally?
   1. Not at all
   2. Only a little
   3. A moderate amount
   4. A great deal
   5. Don’t know
6. Which of the following statements comes closest to your view?
   1. Global warming is not happening.
   2. Humans cannot reduce global warming, even if it is happening.
   3. Humans could reduce global warming, but people are not willing to change their behavior, so we are not going to.
   4. Humans could reduce global warming, but it is unclear at this point, whether we will do what is needed.
   5. Humans can reduce global warming, and we are going to do so successfully.
7. Rate your opinion of the following statement: The actions of a single individual will not make any difference in global warming.
   1. Strongly agree
   2. Somewhat agree
   3. Neutral
   4. Somewhat disagree
   5. Strongly disagree
8. Rate your opinion of the following statement: New technologies can solve global warming, without individuals having to make big changes in their lives.
   1. Strongly agree
   2. Somewhat agree
   3. Neutral
   4. Somewhat disagree
   5. Strongly disagree
9. Which comes closer to your own view?
   1. Most scientists think global warming is happening.
   2. Most scientists think global warming is not happening.
   3. There is a lot of disagreement among scientists about whether or not global warming is happening.
   4. Don't know enough to say.
10. How much do you agree or disagree with the following statement: “I have personally experienced the effects of global warming.”
    1. Strongly agree
    2. Somewhat agree
    3. Neutral
    4. Somewhat disagree
    5. Strongly disagree
11. How many of your friends share your views on global warming?
    1. None
    2. A few
12. I personally recommended COMM 168 to other students
    1. True
    2. False

The following questions relate to your participation in COMM 168: Global Climate Change at San José State University, and if this course has affected any of your actions or decisions since you took the course.

1. As a result of my participation in this course, I have taken the following actions to reduce the amount of waste produced in my home. (Check all that apply):
   - Recycle more often
   - Buy products that have less packaging
   - Compost food scraps or food waste
   - Give away or donate products so that they can be reused
   - Other:
   - The course didn’t have an influence on these actions
2. As a result of my participation in this course, I have taken the following actions to reduce energy consumption at home (Check all that apply):
   - Purchased renewable energy from utilities
   - Purchased energy saving appliances
   - Installed solar P V
   - Installed solar hot water
   - Changed traditional light bulbs to energy-efficient light bulbs
   - Other:
   - The course had no influence on my home energy use
3. As a result of my participation in this course, over the course of a week, I make food choices to reduce carbon emissions
   1. All the time
   2. Often
   3. Sometimes
   4. Occasionally
   5. The course had no influence on my food choices
4. As a result of my participation in this course, I have made the following changes to my transportation methods (Check all that apply):
   - Purchased a hybrid car
   - Carpool regularly
   - Purchased a more gas-efficient car
   - Used public transportation more often
   - Used a bicycle instead of a car as transportation method
   - The course didn’t have an influence on my transportation methods
5. As a result of my participation in this course, I purchase carbon offsets when I fly.
   1. All the time
   2. Often
   3. Sometimes
   4. Occasionally
   5. Never
   6. The course had no influence on my purchase of carbon offsets
6. Was there any other situation when you remember how particular content of the class influenced a decision(s) in your life? Explain.
