# Supplementary material for: The role of climate change education on individual lifetime carbon emissions
Source: PLoS One. 2020 Feb 4;15(2):e0206266. doi: 10.1371/journal.pone.0206266 (PMC6999882; doi:10.1371/journal.pone.0206266)
Supplement: S2 Text — (DOCX) [file pone.0206266.s002.docx]

S2 Text: Procedure for estimating reductions in carbon emissions from the survey responses.

In the survey, participants described particular actions they have taken as a result of taking the COMM 168 class. We use the CoolClimate calculator to estimate how those actions change an individual’s annual carbon emissions. The CoolClimate Network is a partnership between government, business, NGOs and universities including the University of California, Berkeley, and this tool is supported and endorsed by the California Air Resources Board [34].

The categories of questions to estimate the household carbon emissions include travel, home, food, goods and services. As shown in Figure 1 in S2 Text, each of these categories contain subsections that include additional questions that allow for more accurate estimates of carbon emissions for each category. The calculator makes various assumptions based on user responses, but it also allows for more detailed responses through an advanced section. For example, in the housing section, the advanced section allows the user to enter detailed information about the building design (e.g., area and type of insulation including windows) and the type of appliances and heating/cooling systems, versus the regular analysis that estimates carbon emissions based on how much energy an average household uses.


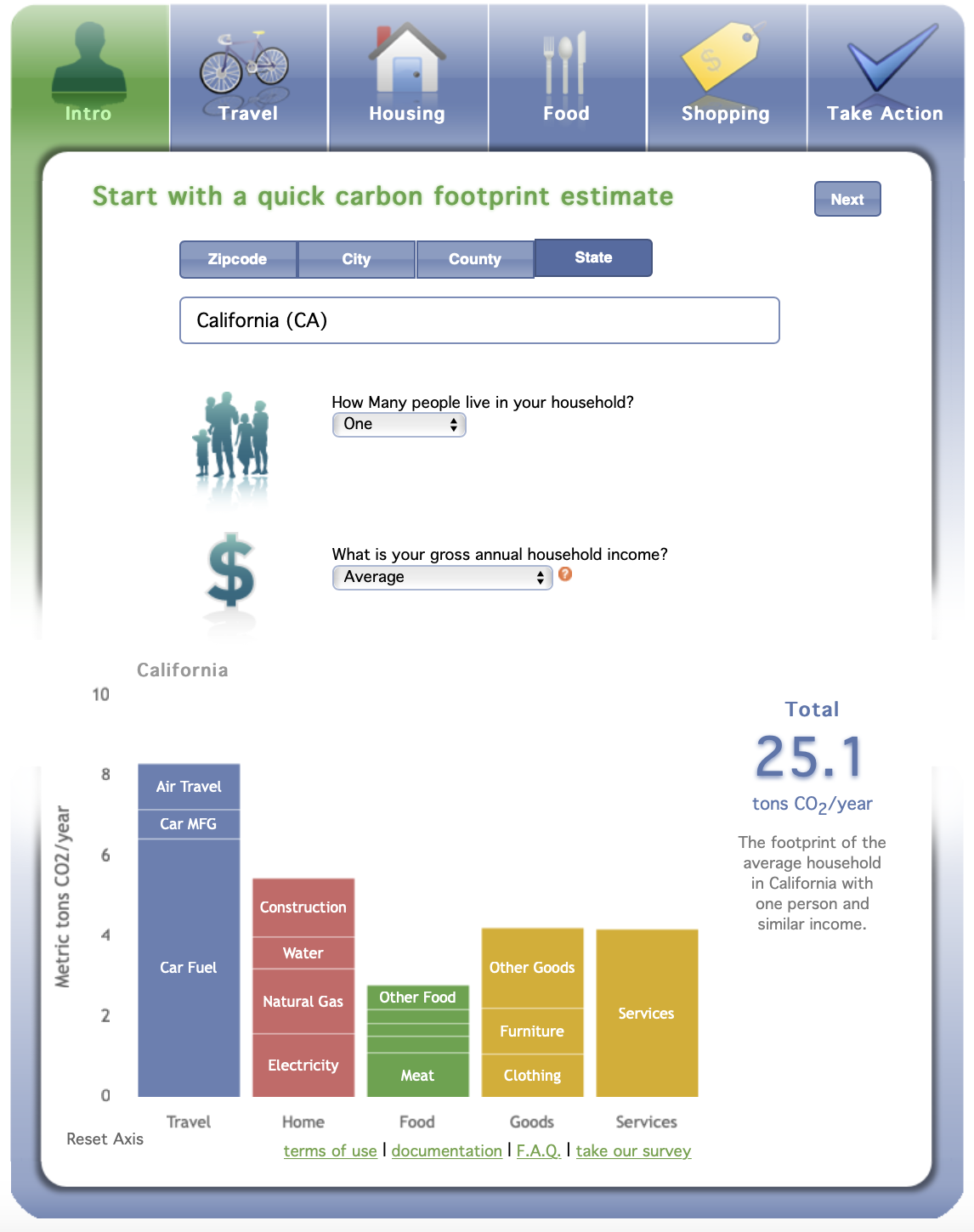


**Figure 1 in S2 Text Screen shot from the CoolClimate calculator developed in part by the California Air Resources Board (**[**www.coolcalifornia.org)**](http://www.coolcalifornia.org))**. Reprinted from the Cool Climate Network under a CC BY license, with permission from Cool Climate Network, original copyright 2019.**

The following procedure is used to estimate the reductions in carbon emissions due to specific actions. We start by selecting one person with an average annual household income for the state of California. Given the internal assumptions based on the categories of travel, home, food, goods and services, the annual carbon emissions are 25.1 tons of CO_2_/year for the individual. We then select the ‘Take Action’ section, where changes in individual actions together with the underlying assumptions can be made for a number of categories. We use this section to match the responses from the COMM 168 participants with reductions of carbon emissions, using a set of assumptions that are shown in Table 1 of S2 Text. Each assumption comes from the CoolClimate calculator and provides an estimate of how a particular action would reduce annual carbon emissions. For example, one of the survey questions asks if the participant purchased renewable energy from their utility. The CoolClimate calculator assumes the household uses 4,396 kWh of electricity/year, which produces 1.36 tons of CO_2_. We assume that participants who answer ‘yes’ to the question about purchasing renewable energy from their utility would get 100% of their electricity from clean energy and thus would save 1.36 tons of CO_2_/year. In another example, for the question about purchasing a more efficient vehicle, the calculator assumes that the individual would trade in a 22 mpg vehicle for a 32 mpg vehicle, and after driving 13,100 miles/year, they would save 2.08 tons of CO_2_/year. Each of the question responses and associated assumptions are given in Table 1 of S2 Text.

**Table 1 in S2 Text. Description of the assumptions and reductions in carbon emissions for each of the survey responses.**

| **Question response** | **CO_2_ reduction (tons/year)** | **Assumptions used in CoolClimate calculator** |
| --- | --- | --- |
| Buy renewable energy | 1.36 | 100% electricity from renewables |
| Buy energy star appliances | 0.04 | Energy star fridge or other products |
| Install solar panels | 0.68 | 50% electricity from renewables |
| Install solar hot water | 0.4 | 50% of heating of water from solar hot water |
| Change light bulbs | 0.16 | 5 bulbs used 5 hours per day |
| Buy hybrid/electric car | 4.22 | average of hybrids (40 mpg*2/3) + electric vehicles (99 mpg*1/3)) = 59.67 mpg |
| Recycle more often, buy products with less packaging, compost food scraps, give away/donate products | 0.42 | Reduce waste by 25% for each action |
| Carpool regularly | 0.85 | 3 times/week |
| Buy more fuel efficient vehicle | 2.08 | 32 mpg vs. 22 mpg for 13,100 miles/yr. |
| Use public transit more | 0.42 | 20 miles/week in bus instead of 22 mpg in car |
| Use bike for transportation | 0.53 | 20 miles/week instead of 22 mpg in car |
| Make food choices to reduce emissions | 0.69 | Response of ‘all the time’ or ‘often’ is default setting for ‘low carbon version of American diet’ (0.69 tons/year). Response of ‘sometimes’ or ‘occasionally’ is a reduction of 0.46 tons/year through increase in meat consumption from 244 calories (default) to 353 calories. |
| Buy carbon offsets for flying | 0.93 | Response of ‘all the time’ or ‘often’ is 80% of flights purchased offsets (0.93 tons/yr.). Response of ‘sometimes’ or ‘occasionally’ is 40% of flights purchased offsets (0.46 tons/yr.). |
