# Supplementary material for: The role of climate change education on individual lifetime carbon emissions
Source: PLoS One. 2020 Feb 4;15(2):e0206266. doi: 10.1371/journal.pone.0206266 (PMC6999882; doi:10.1371/journal.pone.0206266)
Supplement: S3 Text — (DOCX) [file pone.0206266.s003.docx]

S3 Text: Focus group protocol

A small number of subjects (5-10) for the semi-structured interviews will be selected randomly from the list of completed surveys. This follow-up semi-structured interview will be conducted to better understand their survey responses with more detail regarding actual practices and experiences.

Below is the focus group protocol that was used by the research team. AMT is Anne Marie Todd and ECC is Eugene Cordero. Each interview will be recorded, and transcripts will be created so that analysis of the focus groups can be completed.

Participants should be asked if we could use their first names in reference to any of their responses. Otherwise, use a pseudonym and note this in the published work.

Script and guidance for the semi-structured interviews are provided below.

AMT: *Invite people to grab pizza and a drink and sit down.*

AMT: *Open discussion about research project.*

In this research project, alumni of the Global Climate Change course were asked to complete a survey of open-ended questions about course content, their career path, and personal climate change mitigation strategies. In this part of the study, we are following up with focus groups – discussions among a small group of people that will allow us to learn more about the impact of the class.

ECC: *Explain results of survey (not content, but number of responses, broad themes).*

AMT: *Explains the guidelines/structure of the focus group.*

Ask open-ended questions; there is no right answer.

Make sure anyone who wants has a chance to speak.

Is it okay if we record this? Please let us know if you would prefer not to be directly quoted.

Let’s begin!

**Engagement questions (introduce participants and make them comfortable with the topic of discussion)**

What was most memorable part of the course? What was your favorite part of the course?

When you talk about this course, how do you describe this course to colleagues, friends, etc.? How do you explain it?

Why did you sign up for the class?

**Exploration questions (heart of the discussion) (*should be no more than 8 questions)**

How is climate change affecting your life today?

Did the class have any effect on your career trajectory?  If so, can you explain?

Can you provide any particular examples of how the class affected any of your lifestyle choices?

What aspects of the class do you remember the most, or do you feel were the most impactful to you, or to other students in the class?

What actions are you taking to respond to climate change?  Do you think these are helpful?

How important do you think individual actions will be in responding to climate change?

**Exit questions (check to see if anything was missed in the discussion)**

Anything else you’d like to add?

Anything else you think we missed? Any questions we should have asked?

Thank you very much for your participation!
