# Supplementary material for: The role of climate change education on individual lifetime carbon emissions
Source: PLoS One. 2020 Feb 4;15(2):e0206266. doi: 10.1371/journal.pone.0206266 (PMC6999882; doi:10.1371/journal.pone.0206266)
Supplement: S4 Text — (PDF) [file pone.0206266.s004.pdf]

S4 Text: COMM 168 Syllabus

COMM/ENVS/METR 168: GLOBAL CLIMATE CHANGE
A TEAM SJSU STUDIES COURSE: Areas R, S, V, & Z

FALL 2009–SPRING 2010 TR 1:30–4:15 ROOM: DMH 234

INSTRUCTORS:

Dr. Eugene Cordero, Department of Meteorology, DH 618, 924-5188, cordero@met.sjsu.edu
Office Hours: Tues 12:00-1:30, Wed 2:00-4:00pm or by appointment.

Dr. Alexander Gershenson, Environmental Studies, WSQ 115, 924-5427, envs168@gmail.com
Office Hours: TBA

Dr. Anne Marie Todd, Communication Studies, HGH 205, 924-5391, amt@sjsu.edu
Office hours: Tues. 11:45-1:30, 4:30-6; Thurs. 11:45-1:30, and by appointment.
**Note: beginning November 3, my Tuesday 4:30-6 p.m. office hours will change to Thursday, 9-10:30 a.m. for the rest of the semester.*

COURSE DESCRIPTION:
Many different scientific observations and measurements indicate that Earth is experiencing global-scale changes in climate, i.e., in the long-term distributions of temperature, cloud cover, precipitation, and extreme weather events. Scientific consensus considers at least some of these changes to be caused or accelerated by human activities. The economic, ecological, social, and cultural challenges caused by global climate change will affect everyone on the planet, and are very likely to have disproportionate impacts on developing nations. Global climate change is an urgent issue that requires educated and engaged citizens to raise awareness and take mitigating actions. In this course, we will study global climate change from an interdisciplinary perspective, incorporating scientific and social scientific approaches to understanding processes and effects. We will study the socioeconomic contexts of environmental effects and how globally diverse cultural perspectives influence strategies to mitigate climate change effects.

A note about this course (Team SJSU Studies): This is a year-long course: you take 6 units in Fall 2009 and 3 units in Spring 2010. You will receive GE credit for Areas R, S, V, and Z after you have successfully completed all 9 units of the course. **In order to receive GE credit, you must receive a grade of "C" or better in the 3-unit portion of the course (Spring 2010). A grade of a "C-" will not earn GE credit.**

This course is team-taught. We meet for extended class periods. We will cover *a lot* of material on numerous topics and engage in various activities related to global climate change and the learning objectives of the four areas of SJSU Studies. Assignments, readings, and class discussions and activities are designed to help you recognize connections among concepts from many different disciplines, and to critically evaluate and integrate them as part of a life-long learning process about global climate change. This course will help students to live and work intelligently, responsibly, and cooperatively in a multicultural society and to develop abilities to address complex issues using disciplined analytical skills and creative techniques.

PREREQUISITES: A grade of "C" or better in English IB, passage of the Writing Skills Test (WST), upper-division standing, and completion of Core GE.

COURSE POLICIES:

* This is a participation-intensive course that relies on your consistent and active engagement. In case of an emergency, please do everything in your power to contact us prior to missing class. We will only accept late work in cases of extreme personal emergency; furthermore, such work may be subject to a 50% grade penalty and requires some level of documentation.

* We will regularly use the course's Blackboard website [http://sjsu6.blackboard.com/webct/] for announcements, reading assignments, copies of handouts, and uploads of instructor presentations. You are responsible for regularly (daily, perhaps) checking the course's Blackboard page.

* If you need course adaptations or accommodations because of a disability, or if you need special arrangements in case the building must be evacuated, please make an appointment with one of the professors as soon as possible, or see us during office hours. Presidential Directive 97-03 requires that students with disabilities requesting accommodations must register with DRC to establish a record of their disability.

* Your own commitment to learning, as evidenced by your enrollment at San José State University, and the University's Academic Integrity Policy requires you to be honest in all your academic course work. Faculty members are required to report all infractions to the Office of Student Conduct and Ethical Development. The policy on academic integrity can be found at http://sa.sjsu.edu/student_conduct. You should, therefore, submit your own, original work for this course. We will uphold San José State University's policy on academic integrity. Consequently, an instance of academic misconduct (e.g., plagiarism, cheating, taking credit for the work of others, submitting work for another course as work for this one, etc.) will likely result in a failing course grade.

* Computers in the classroom: Although the use of computers can be useful for taking notes, they can also be extremely distracting for nearby students when the user is browsing the internet, instant messaging etc. Certainly, this compromises the learning environment our of course. We therefore will not allow laptop use in the classroom unless a student formally requests permission with a letter certifying that computer use will be strictly for note taking. If a student violates the conditions of use, permission to use the computer will be revoked.

*Other electronic devices, such as cell phones, music players, etc. are to be turned off or completely silenced during the duration of class, as they are a needless distraction for the entire class.

REQUIRED TEXTS:

Dauncey, G., & Mazza, P. (2001). *Stormy weather: 101 solutions to global climate change.* Gabriola Island, British Columbia: New Society Publishers.

Kirszner, L. & Mandell, S. (2008). *The pocket handbook* (4th Ed.). Belmont: Wadsworth.

Mann, M., & Kump, L. (2009). *Dire predictions: Understanding global warming.* New York, DK Publishing.

**OTHER READINGS WILL BE ASSIGNED VIA THE COURSE WEBSITE. IT IS YOUR RESPONSIBILITY TO STAY CURRENT WITH INFORMATION ON THE WEBSITE AND COMPLETE READINGS ON TIME.

LEARNING OBJECTIVES & ASSESSMENT:
Learning objectives are developed to assist students in understanding the main goals and expectations of the course. Teaching and learning activities are designed with these objectives in mind while assessment activities help us measure student achievement of these objectives. This course will incorporate writing assignments throughout the two semesters, and will meet the requisite 17,000 words required of the four SJSU Studies areas. Some written assignments will be adaptable to students' specific disciplines.

Assessment is designed to determine how well students have achieved the goals of the learning objectives and thus form an important component of the course. Each student will be assessed through a combination of writing assignments, exams, and course projects. Each assignment is linked to the student learning objectives (SLO) noted in the greensheet by Area and SLO number (ex: Riii). Students will complete diagnostic, midterm, and summative assessment rubrics each semester in addition to written reflection and evaluation of their own work.

The Area R (Earth and Environment) General Education learning objectives are:
- A student should be able to demonstrate an understanding of the methods and limits of scientific investigation.
- A student should be able to distinguish science from pseudo-science.
- A student should be able to apply a scientific approach to answer questions about the earth and environment.

R: The specific learning objectives in this area for this course are:
i. To develop an understanding of the fundamental processes responsible for past and present climate change.
ii. To understand and be able to articulate the implications and uncertainties related to our changing climate.
iii. To understand the connection between consumption, energy use, and global climate change.
iv. To be able to discuss and evaluate various climate change mitigation strategies.
v. To be able to understand and evaluate the evidence for past and present climate change.

The Area S (Self, Society & Equality in the U.S.) General Education learning objectives are:
- To be able to describe how identities (i.e. religious, gender, ethnic, racial, class, sexual orientation, disability, and/or age) are shaped by cultural and societal influences within contexts of equality and inequality.
- To be able to describe historical, social, political, and economic processes producing diversity, equality, and structured inequalities in the U.S.
- To be able to describe social actions which have led to greater equality and social justice in the U.S. (i.e. religious, gender, ethnic, racial, class, sexual orientation, disability, and/or age).
- To recognize and appreciate constructive interactions between people from different cultural, racial, and ethnic groups within the U.S.

S: The specific learning objectives in this area for this course are:
i. To be able to describe how cultural and societal effects of climate change shape the identities of individuals and communities.
ii. To be able to describe the processes of the fossil fuel economy that creates structured inequalities in the United States.

iii. To be able to identify climate change mitigation strategies and describe actions that can lead to environmental justice in the U.S.
iv. To recognize and appreciate constructive interactions between people from different cultural, racial, and ethnic groups in the U.S., and to apply this knowledge to conduct a community needs assessment and develop a community outreach strategy regarding climate change.

The Area V (Culture, Civilization & Global Understanding) General Education learning objectives are:

- To be able to compare systematically the ideas, values, images, cultural artifacts, economic structures, technological developments, and/or attitudes of people from more than one culture outside the U.S.
- To be able to identify the historical context of ideas and cultural traditions outside the U.S. and how they have influenced American culture.
- To be able to explain how a culture outside the U.S. has changed in response to internal and external pressures.

V. The specific learning objectives in this area for this course are:

i. To be able to compare international policy responses and cultural perceptions of climate change.
ii. To be able to compare policy mechanisms, economic development patterns, and governance structures that influence national and cultural responses toward international efforts to mitigate adverse impacts of climate change.
iii. To be able to identify how international policy actions are affected by historical, cultural, and economic contexts of developed and developing countries, with emphasis on how international cultural perspectives affect the United States' response.
iv. To be able to explain how the cultures of developing countries have responded to international negotiations of climate change.

The Area Z (Written Communication II) General Education learning objectives are:

- To be able to write complete essays that demonstrate college-level proficiency.
- To be able to refine the competencies established in Written Communication IA and IB.
- To be able to express (explain, analyze, develop, and criticize) ideas effectively, including ideas encountered in multiple readings and expressed in different forms of discourse.
- To be able to organize and develop essays and documents for both professional and general audiences, including appropriate editorial standards for citing primary and secondary sources.

Z: The specific learning objectives in this area for this course are:

i. To be able to write complete essays on the importance of climate change for specific disciplines.
ii. To be able to explain and analyze public attitudes and public discourse about climate change effectively.
iii. To be able to research, organize, and develop documents on various aspects of climate change policy relevant to general and professional audiences.
iv. To be able to express ideas about the processes and effects of climate change effectively for community audiences.

ASSIGNMENTS AND GRADING:

- For most written assignments, "on-time" completion includes not only submission of a hard copy in class, but also submission of a computer file to www.turnitin.com by the assigned deadline. You must have an account at turnitin. Our *class ID* is 2815262, the password is "carbon". We will discuss the details of this policy at our second class meeting; please contact us if you have any questions.

- There will be spontaneous writing activities throughout the course. These are in-class writing assignments, which all students are responsible for completing as part of the revision and feedback process of that particular writing assignment.

- Letter grades will be assigned according to the following point scale:

A	925-1000 points	C	725-764 points
A-	895-924 points	C-	695-724 points
B+	865-894 points	D+	665-694 points
B	825-864 points	D	625-664 points
B-	795-824 points	D-	595-624 points
C+	765-794 points	F	0-594 points

FALL 2009 ASSIGNMENTS

Short Paper #1: 15%. 1000 words. Required revision: 1000 words. (SLO: Zi) Prof. Todd
Compose an essay about the importance of climate change for your specific discipline. You should consult at least two sources from your field. Using these articles, compose an organized essay, addressed to an audience of peers in your field, articulating at least three reasons why the issue of climate change is important to your discipline. *A detailed assignment sheet and grading rubric will be handed out and discussed in class.*

Ecologial Footprint Project: 10%. 1000 words. (SLO: Riii, Riv) Prof. Cordero
Students will compute their ecological or carbon footprint based on their personal lifestyle. Students will then answer a variety of questions about their footprint and their reaction to this activity. In addition, students will be required to simulate a lower footprint lifestyle for a short period of time. During this time, student will work in small groups to share strategies for effectively reducing their footprint. *A detailed assignment sheet and grading rubric will be handed out and discussed in class.*

Exam #1: 10%. 500 words (SLO: Ri, Rii, Riii, Riv, Rv)

Exam #2: 10%. 500 words (SLO: Ri, Rii, Riii, Riv, Rv)

Exam #3 (Final): 10%. 500 words (SLO: Ri, Rii, Riii, Riv)

Short Paper #2: 15%. 1000 words. Required revision: 1000 words. (SLO: Riv, Sii, Siii, Vii, Viii, Zii) Prof. Gershenson
Students will examine the debate surrounding the Waxman-Markey Bill using news media and editorial sources. Students will examine the evolution of ideas and the legislative progress of the current US Climate Bill, and compare it to past efforts. *A detailed assignment sheet and grading rubric will be handed out and discussed in class.*

Research Paper Topic Proposal: 5%. 500 words. Required revision: 500 words. (SLO: Ziii)
Your research paper will be submitted in spring semester, but you will begin this semester. Write

a topic proposal for your research paper, which will be turned in the spring semester. You may elaborate on the list of suggested topics or develop your own idea. *A detailed assignment sheet and grading rubric will be handed out and discussed in class.*

Research Paper Annotated Bibliography: 5%. 1000 words (SLO: Ziii)
Each student will compile an annotated bibliography of 4-5 articles on their topic. *A detailed assignment sheet and grading rubric will be handed out and discussed in class.*

In class activities: 20%. 1500 words. Various activities will be assigned and completed in class.

<u>SPRING 2010 ASSIGNMENTS</u>

Research Paper: 30%. Rough Draft: 2000 words, Required Revision 3000 words. (SLO: Si, Sii, Siii, Siv, Vi, Vii, Viii, Viv, Zii, Ziii)
You will write up a research paper based on your topic proposal and research conducted for your annotated bibliography. *A detailed assignment sheet and grading rubric will be handed out and discussed in class.*

Exam #1: 10%. 500 words. (SLO: Ri, Rii, Riii, Riv, Rv)

Community Project: 30%. 2000 words. This assignment is designed to apply the findings of your research project and encourage your interaction with a community regarding climate change. This is a group *action* project. You will work with a group of your peers to engage the community on some aspect of climate change. As a group, you will submit a proposal, a report, and a presentation. Your final community project grade will be a combination of individual and group grades. *A detailed assignment sheet and grading rubric will be handed out and discussed in class.* (SLO: Riii, Riv, Si, Sii, Siii, Siv, Vi, Vii, Viii, Zii, Ziii, Ziv).

Exam #2 (Final): 10%. 500 words. (SLO: Riv, Si, Sii, Siii, Siv, Vi, Vii, Viii, Viv).

In class activities: 20%. 1000 words. Various activities will be assigned and completed in class.

Grading Rubric for Written Assignments

All take-home writing assignments will be graded according to the rubric described below. As specified in the guidelines for GE courses, we will assess not only the content but also the quality and clarity of your writing. Note that the two components are equally weighted.

score	~grade	Content criteria
50%	A+	Outstanding response with superior supporting examples or evidence; unusual insights, creative and original analysis, reasoning, and explanation; superior mastery of content; goes well beyond minimum required for the assignment.
40%	B+	Good, solid response that uses excellent supporting examples or evidence; excellent reasoning and explanations; goes beyond the minimum required for the assignment.
30%	B-	Good, solid response that meets minimum required by assignment. Reasoning and explanations are adequate.
20%	C-	Response is accurate but cursory, and does not meet the minimum required for completeness; some inaccuracies or reasoning flaws; response is too general, lacks specific evidence.
10%	D	Response doesn't effectively address the question; response fails to support assertions with data or examples; major flaws in reasoning; explanations are unclear; displays inadequate understanding of content.
0%	F	Response is missing or not submitted, or does not address the question.

score	~grade	Writing criteria
50%	A	Meets criteria for 40%, plus demonstrates superior grammatical correctness and sense of personal style. Effortlessly readable prose.
40%	B+	Very effective organization of paragraphs and paper; interesting, varied sentences; good grammar (usage, punctuation, etc.); few spelling mistakes; does not read like a first draft.
30%	C+	Reasonably effective organization of paragraphs and paper; serviceable prose; numerous errors of grammar or spelling; reads like a first draft.
20%	C-	Structurally disorganized; paragraphs lack topic sentences or are not developed effectively; awkward sentence structure; poor grammar; poor spelling.
10%	D-	Similar to 20%, but even harder to read.

FALL 2009 COURSE CALENDAR (subject to change)
Reading assignments are on the website. It is your responsibility to do the assigned reading before class.

Tues 25 Aug
Introduction to the course

Thur 27 Aug
Paper #1 Assigned
Greenhouse: Earth & Mars
Projected changes in climate; Mitigation & adaptation measures

Tues 1 Sept
Earth's Energy Balance I
Communication Fundamentals/Writing for your discipline

Thur 3 Sept
Earth's Energy Balance II
Writing discussion

Tues 8 Sept
Paper #1 due
Clouds & Climate I
The biosphere: Biomes and Ecosystems

Thur 10 Sept
Carbon Cycle
Story of Stuff (film)

Tues 15 Sept
Biology overview/introduction
Communicating Climate Change

Thur 17 Sept
Natural Climate Forcing
Climate and Resources

Tues 22 Sept: FURLOUGH DAY

Thur 24 Sept
Rewrite Paper #1 Due
Climate Feedbacks
Review session for exam

Tues 29 Sept
Midterm #1
Writing Discussion

Thur 1 Oct
Recent Climate Change
Land Use and Climate Change

Tues 6 Oct
Intro to Climate Change Impacts
Risk Communication

Thur 8 Oct
Paper #2 Assigned
Aerosols & Radiative Forcing I
Analysis of Climate Change Bill

Tues 13 Oct
History of U.S. Policy I
Aerosols & Radiative Forcing II

Thur 15 Oct
History of U.S. Policy II
Local/Global Audiences

Tues 20 Oct
Paper #2 due
Topic Paper Assigned
Kyoto Protocol
Discussion of Research/Community Project.

Thur 22 Oct
An Inconvenient Truth (film)

Tues 27 Oct: FURLOUGH DAY

Thur 29 Oct
Rewrite of paper #2 due
Climate Models and Future Projections
Review session for exam

Tues 3 Nov
Exam #2
Ecological Footprint Assigned

Thur 5 Nov
Research I

Tues 10 Nov
Topic Paper due
Annotated Bibliography Assigned
Research II
Energy and Climate Change Mitigation

Thur 12 Nov
IPCC: intro, scenarios, products
Food Climate Connections

Tues 17 Nov

Annotated Bibliography Discussion

Thur 19 Nov
Ecological Footprint Due
Projected Impacts II

Tues 24 Nov: FURLOUGH DAY

Thur 26 Nov: NO CLASS — THANKSGIVING

Tues 1 Dec
Climate Connection: Ozone Depletion
Intro to adaptation and mitigation

Thur 3 Dec
Annotated Bibliography Due
Communicating Impacts of Climate Change
Climate Connection: Water

Tues 8 Dec
Discussion, review

Final Exam:
Tuesday, December 15, 1215-1430 OR Monday, December 14, 1445-1700 (TBD)

Please save this greensheet for Spring 2010 so we can reduce copies.
